# Genetic factors, adherence to healthy lifestyle behaviors, and risk of bladder cancer

**DOI:** 10.1186/s12885-023-11455-4

**Published:** 2023-10-12

**Authors:** Qiangsheng He, Siqing Wu, Ying Zhou, Yuchen Liu, Bin Xia, Wenjing Li, Jinyu Zhao, Ningning Mi, Peng Xie, Xiwen Qin, Jinqiu Yuan, Yihang Pan

**Affiliations:** 1 Scientific Research Center, Big Data Center, The Seventh Affiliated Hospital, Sun Yat-Sen University, Shenzhen, 518107 Guangdong China; 2 Clinical Research Center, The Seventh Affiliated Hospital, Sun Yat-Sen University, Shenzhen, 518107 Guangdong China; 3 Guangdong Provincial Key Laboratory of Gastroenterology, Center for Digestive Disease, The Seventh Affiliated Hospital, Sun Yat-Sen University, Shenzhen, 518107 Guangdong China; 4 School of Medicine, Sun Yat-Sen University, Shenzhen, Guangdong 518107 China; 5 Primary Care Office, The Seventh Affiliated Hospital, Sun Yat-Sen University, Shenzhen, 518107 Guangdong China; 6 Department of General Surgery, The First Hospital of Lanzhou University, Lanzhou, 730000 Gansu China; 7 Department of Pharmacology and Pharmacy, LKS Faculty of Medicine, The University of Hong Kong, Pokfulam, Hong Kong; 8 School of Population and Global Health, Faculty of Medicine, Density and Health Sciences, University of Western Australia, Perth, AU-WA Australia

**Keywords:** Genetic risk, Polygenic risk score, Healthy lifestyle, Bladder cancer

## Abstract

**Background:**

Genetic and lifestyle factors both contribute to the pathogenesis of bladder cancer, but the extent to which the increased genetic risk can be mitigated by adhering to a healthy lifestyle remains unclear. We aimed to investigate the association of combined lifestyle factors with bladder cancer risk within genetic risk groups.

**Methods:**

We conducted a prospective study of 375 998 unrelated participants of European ancestry with genotype and lifestyle data and free of cancer from the UK biobank. We generated a polygenic risk score (PRS) using 16 single nucleotide polymorphisms and a healthy lifestyle score based on body weight, smoking status, physical activity, and diet. Cox models were fitted to estimate the hazard ratios (HRs) and 95% confidence intervals (CIs) of genetic and lifestyle factors on bladder cancer.

**Results:**

During a median follow-up of 11.8 years, 880 participants developed bladder cancer. Compared with those with low PRS, participants with intermediate and high PRS had a higher risk of bladder cancer (HR 1.29, 95% CI 1.07–1.56; HR 1.63, 95% CI 1.32–2.02, respectively). An optimal lifestyle was associated with an approximately 50% lower risk of bladder cancer than a poor lifestyle across all genetic strata. Participants with a high genetic risk and a poor lifestyle had 3.6-fold elevated risk of bladder cancer compared with those with a low genetic risk and an optimal lifestyle (HR 3.63, 95% CI 2.23 –5.91).

**Conclusions:**

Adhering to a healthy lifestyle could substantially reduce the bladder cancer risk across all genetic strata, even for high-genetic risk individuals. For all populations, adopting an intermediate lifestyle is more beneficial than a poor one, and adhering to an optimal lifestyle is the ideal effective strategy for bladder cancer prevention.

**Supplementary Information:**

The online version contains supplementary material available at 10.1186/s12885-023-11455-4.

## Background

Bladder cancer is one of the most common incident cancers worldwide, with approximately 600 000 new cases annually reported, leading to over 200 000 deaths each year [1]. Environmental factors, such as tobacco smoking and occupational exposure to carcinogens, are the leading risk factors for bladder cancer [2]. In addition, evidence from family history studies and genome-wide association studies (GWAS) supported the pivotal role of genetics in the development of bladder cancer [3, 4]. A cohort study among Nordic twins indicated that the heritability of bladder cancer was approximately 30% [5]. To date, large-scale GWASs have identified 15 independent loci associated with the risk of bladder cancer in European population [6]. These genetic variants, when combined into a polygenic risk score (PRS), can efficiently predict bladder cancer risk [7, 8]. Several PRSs for bladder cancer have constructed among participants of European ancestry [7–9]. For example, a previous study based on 14 SNPs identified in participants of European ancestry reported that individuals in the highest PRS quintile had a greater than twofold risk of bladder cancer, compared with those in the lowest PRS quintile [7]. Another study using 10 SNPs identified in Caucasian subjects reported that participants with high genetic risk had a 1.52 times higher risk of bladder cancer [9].

Modifiable lifestyle risk factors are the leading contributors to global cancer incidence and mortality [10]. Potentially modifiable lifestyle factors, including smoking, obesity, physical inactivity, and unhealthy dietary pattern have been associated with a higher risk of bladder cancer [2, 11]. For example, evidence based on the BLadder cancer Epidemiology and Nutritional Determinants (BLEND) study has shown that the Mediterranean diet, tea consumption, vegetable intake, and yogurt consumption may be protective against bladder cancer, while the Western dietary pattern and coffee consumption may be risk factors [12–17]. Given that these lifestyle factors often coexist, accumulating recent studies have examined the combined impact of lifestyle factors on cancer risk, and showed that adopting a healthy lifestyle, could markedly decrease the risk of overall cancer, breast, stomach and colorectal cancers [18–21]. However, limited studies investigating the association of combined lifestyle factors with bladder cancer risk currently exist [22].

There is substantial evidence suggesting that adhering to a healthy lifestyle could attenuate the impact of genetic factors on the risk of several cancers, such as cancers of the stomach, prostate and colorectum [19, 21, 23]. For example, a recent study found that individuals at high genetic risk of overall cancer may benefit from adopting a healthy lifestyle [18]. However, no study to date has investigated the joint effects of genetic variants and combined lifestyle factors on bladder cancer risk. To what extent individuals with an increased genetic risk of bladder cancer can be offset by adhering to a healthy lifestyle remains unknown.

Based on the UK Biobank cohort, we aimed to evaluate the extent to which adhering to a healthy lifestyle might mitigate the risk of bladder cancer among individuals with a different genetic risk, particularly among individuals at a high genetic risk defined by the PRS.

## Methods

### Study population

The UK Biobank is a large ongoing population-based prospective cohort of over 500 000 individuals aged 37–73 years. Between 2006 and 2010, eligible participants were invited to attend one of 22 assessment centers throughout England, Scotland, and Wales. During the baseline assessment, participants were asked to complete a touchscreen questionnaire and a brief interview to collect sociodemographic, lifestyle factors and health-related information. Participants also underwent a range of physical measurements and provided biological samples. More details of UK Biobank could be found elsewhere [24]. Each participant provided written informed consent before data collection. The UK Biobank study has been approved by the North West Multi-centre Research Ethics Committee.

In the present study, we included 488 169 participants with available genetic data and excluded participants of non-European ancestry or related individuals (*n* = 69 790), those with a prior cancer diagnosis (*n* = 31 237), those with incomplete data on lifestyle factors (*n* = 10 223), and those who subsequently withdrew from the study (*n* = 921). Figure S1 in the Supporting Information showed the flowchart of the study sample selection.

### Ascertainment of bladder cancer

In the UK Biobank, participants were followed through records linkage to the Health and Social Care Information Centre (in England and Wales) and the National Health Service Central Register (in Scotland). These registrations recorded the diagnosis of cancer and cancer deaths using the 10th revision of the international classification of diseases (ICD-10) codes. The primary outcome of the study was incident bladder cancer (ICD 10 C67), or bladder cancer listed as the underlying cause of death on the death certificate.

### Assessment of exposure and covariates

At baseline, information on sociodemographic characteristics (age, sex), lifestyle habits (smoking status, physical activity, and dietary intake) and health and medical history, were obtained using a self-administered touchscreen questionnaire and nurse-led interviews. Physical activity was assessed using the validated Short International Physical Activity Questionnaire (IPAQ). Height, weight and waist circumference (WC), were measured by trained research staff using standardized procedures. More details of these measures can be found elsewhere [24].

### Healthy lifestyle score

We created a healthy lifestyle score (HLS) based on the new World Cancer Research Fund/American Institute of Cancer Research (WCRF/AICR) lifestyle score [25]. Specifically, the HLS was generated from the combination of four lifestyle factors, including body weight, smoking status, physical activity, and diet score (see Supporting Information Table S1). According to WCRF guidelines, each component was categorized into 3 groups (optimal, intermediate, poor). Body weight was classified according to an individual’s BMI and WC. Optimal weight was defined as 18.5 ≤ BMI < 25 kg/m^2^ as well as WC < 94 cm in men and WC < 80 cm in women, poor weight as BMI ≥ 30 kg/m^2^, or WC ≥ 102 cm in men and WC ≥ 88 cm in women, or intermediate (all other combinations). Smoking status was defined as optimal if participants never smoked or quit 10 years ago, intermediate if they were previous smokers, or as poor if they were current smokers. Physical activity was defined as optimal if individuals had ≥ 150 min/week per week moderate or ≥ 75 min per week vigorous or an equivalent combination, intermediate as 1– 149 min/week moderate or 1–74 min/week vigorous or 1–149 min/week mixed activity, and poor as not performing any moderate or vigorous activity. A diet score was built based on the sum of intake of fruits and vegetables, whole grains, red and processed meat, and was then categorized into 3 groups based on tertiles (optimal, intermediate, poor). Given that the greater consumption of tea could reduce the risk of bladder cancer based on WCRF/AICR report [26], we added the tea intake as a new component to the diet score. The healthy lifestyle was categorized into optimal (having at least 3 optimal lifestyle factors), poor (having at least 3 poor lifestyle factors), or intermediate (all other combinations).

We also constructed a weighted healthy lifestyle score based on the β coefficients of each lifestyle factor in the Cox model for bladder cancer risk as a sensitivity analysis. The weighted healthy lifestyle score was derived by summing the product of each lifestyle factor with its corresponding β coefficient, then dividing the sum by the total of the β coefficients, and subsequently categorizing it into three groups based on tertiles (optimal, intermediate, poor).

### Genotyping and imputation

Details of the array design, genotyping process and quality control in the UK Biobank can be found elsewhere [27]. In brief, 488 170 participants had DNA samples assayed using two genotyping arrays sharing a 95% marker content, the UK Biobank Axiom array (825 927 markers) and the UK BiLEVE Axiom Array (807 411 markers in 49 934 participants) [27]. Genotype data was phased and imputed with SHAPEIT3 and IMPUTE3 based on reference panels of the Haplotype Reference Consortium or UK10K and 1000 Genomes phase 3. From the resulting dataset, we excluded participants with a mismatch between reported and genetic sex, sex chromosome aneuploidy, a high missingness or excess heterozygosity, participants of non-European ancestry, second-degree (or higher) related participants (kinship coefficient ≥ 0.088).

### Polygenic risk score

In previous published studies, a PRS for bladder cancer was built based on the 15 SNPs identified in previous GWAS among individuals of European ancestry [7, 8, 18, 28]. Using a similar approach, we searched and reviewed the previous published GWAS of bladder cancer to identify susceptibility SNPs. A total of 16 SNPs with minor allele frequency (MAF) ≥ 0.01 and *P* < 5 × 10^−8^ were used to construct the PRS in our study (Supporting Information Table S2). The corresponding effect estimates were extracted from the largest studies accordingly. For risk variants that were not available in UK Biobank data, the proxy SNPs (r^2^ > 0.8, reference population: EUR) were identified using the LDlink (https://ldlink.nci.nih.gov/) [29]. We excluded SNPs identified exclusively from non-European populations, with allele mismatches or in high linkage disequilibrium (LD; r^2^ ≥ 0.2), and palindromic SNPs with MAF ≥ 0.45 [28, 30].

PRS for each study participant was constructed by summing the product of the number of risk alleles (0, 1, and 2) for each SNP and its respective weight [30]. The PRS was then categorized into low (the bottom quintile of PRS), intermediate (quintiles 2–4), and high (the top quintile).

### Statistical analysis

We calculated person-years from the recruitment date to the date of the first diagnosis of any cancer, death, or the last date of follow-up (31 December 2020), whichever came first. Multivariable Cox proportional hazards models were fitted to evaluate the effects of genetic factors, lifestyle factors, and the combination of genetic and lifestyle factors on bladder cancer risk, by calculating HRs and 95% confidence intervals (CI). To evaluate the extent to which adhering to a healthy lifestyle might mitigate the underlying bladder cancer risk owing to genetic susceptibility, we created joint categories of the genetic and lifestyle factors categories, with the high-risk group (i.e., with high genetic risk and poor lifestyle) as the reference group. Cox models were adjusted for age, sex, family history of cancer, socioeconomic status, and the first 10 principal components of ancestry. The interaction between the lifestyle and genetic risk score was tested by including an interaction term in the regression model. We further calculated absolute risk difference over a 10-year period, and the number needed to adopt an optimal lifestyle to prevent one bladder cancer case in 10 years, based on the method described by Altman D.G and Andersen P.K [31]. We also calculated the population-attributable fraction to estimate the proportion of bladder cancer cases that would be prevented if the whole population were in the optimal lifestyle category.

Given that there is considerable gender difference in the incidence of bladder cancer, we repeated all analyses separately by sex. We conducted several sensitivity analyses to test the robustness of the primary results. Firstly, to minimize the possibility of the reverse causality bias resulting from lifestyle changes caused by undiagnosed cancer, we excluded events that occurred within the first 2 years of follow-up. Secondly, we limited our analyses to the participants of white British descent. Thirdly, we used the subdistribution hazard model approach proposed by Fine and Gray to account for the competing risk of death, and diagnosis of any other cancer (except for non-melanoma skin cancer). Fourthly, we created a weighted healthy lifestyle score to control for varied effect estimates of associations between different lifestyle factors and bladder cancer. Fifth, we developed a new lifestyle score without smoking status to identify whether smoking status might drive the associations between lifestyle with bladder cancer.

## Results

A total of 375 998 participants were included in the final analysis (Supporting Information fig S1). Table 1 presented the baseline characteristics of the study population. In total, 42.5% participants reported adopting an optimal overall lifestyle, 49.9% adopting an intermediate overall lifestyle, and 7.6% adopting a poor lifestyle. In general, individuals with poor lifestyle tended to be female and less deprived. The PRS was not associated with any of the lifestyle factors except for smoking status (Supporting Information Table S3). During a median follow-up of 11.8 years, 880 incident bladder cancer was identified.

**Table 1:** Baseline characteristics of participants by lifestyle categories

	Poor	Intermediate	Optimal
Participants	28466	187840	159692
Age (years)	56.3 (7.89)	57.0 (7.97)	57.2 (8.01)
Gender			
Female	13193 (46.3)	94370 (50.2)	91640 (57.4)
Male	15273 (53.7)	93470 (49.8)	68052 (42.6)
Family history of cancer			
No	18306 (64.3)	120169 (64.0)	103193 (64.6)
Yes	10160 (35.7)	67671 (36.0)	56499 (35.4)
Highest fifth of index of multiple deprivation quintile	8637 (30.3)	36289 (19.3)	20998 (13.1)
Healthy weight			
Poor	22704 (79.8)	93186 (49.6)	20451 (12.8)
Intermediate	4904 (17.2)	70377 (37.5)	61927 (38.8)
Optimal	858 (3.0)	24277 (12.9)	77314 (48.4)
Smoking			
Poor	14046 (49.3)	21540 (11.5)	1949 (1.2)
Intermediate	8075 (28.4)	44774 (23.8)	17686 (11.1)
Optimal	6345 (22.3)	121526 (64.7)	140057 (87.7)
Physically active			
Poor	15733 (55.3)	27322 (14.5)	2597 (1.6)
Intermediate	9946 (34.9)	84266 (44.9)	33519 (21.0)
Optimal	2787 (9.8)	76252 (40.6)	123576 (77.4)
Diet			
Poor	24934 (87.6)	87756 (46.7)	9865 (6.2)
Intermediate	2966 (10.4)	52724 (28.1)	30595 (19.2)
Optimal	566 (2.0)	47360 (25.2)	119232 (74.7)
Genetic Risk			
Low	5741 (20.2)	37853 (20.2)	32210 (20.2)
Intermediate	16837 (59.1)	111348 (59.3)	94874 (59.4)
High	5888 (20.7)	38639 (20.6)	32608 (20.4)

### Associations of PRS with bladder cancer

**Fig. 1:**
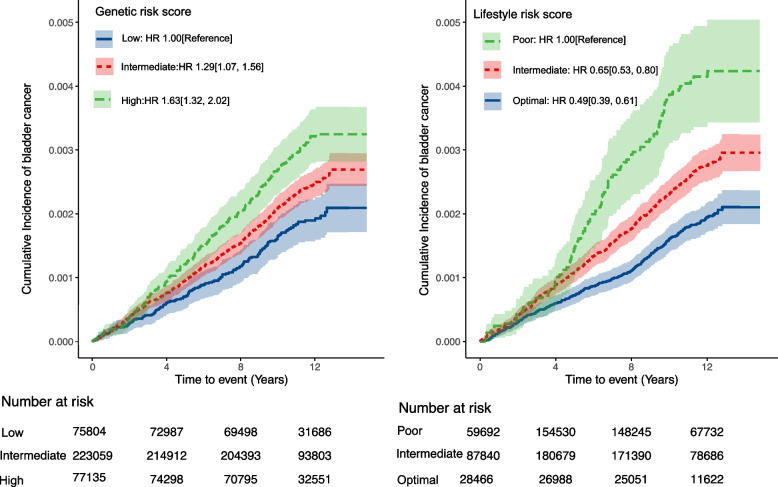
Cumulative risk of incident bladder cancer according to genetic and lifestyle categories. Abbreviation: HR, hazard ratio. Estimated effects were based on Cox proportional hazards regression models, adjusted for age, sex, socioeconomic status (index of multiple deprivation, fifth), family history of cancer, first 10 principal components of ancestry, and additionally mutually adjusted for genetic risk and healthy lifestyle categories

As shown in the Fig. 1 and Table 2, higher PRS was significantly associated with an increased risk of bladder cancer (HR 1.20, 95% CI 1.13–1.29 per SD increment). Compared with those with low PRS (the lowest quintile of PRS), participants with intermediate (quintiles 2 to 4) and high PRS (the highest quintile) had a higher risk of incident bladder cancer (HR 1.29, 95% CI 1.07–1.56; HR 1.63 95% CI 1.32–2.02, respectively). The association was unchanged after additional adjustment for lifestyle factors, indicating that genetic risk was independently associated with bladder cancer risk regardless of lifestyle factors. The similar pattern of results was noted when regrouping the PRS into tertiles or quartiles (Supporting Information Table S4), or restricting the analysis to participants of white British descent, or in the competing risk analysis using Fine-Gray subdistribution hazard model (Supporting Information Table S5).

**Table 2:** Association of genetic and lifestyle factors with bladder cancer risk^a^

	Cases/ Person-years	Hazard ratio (95% CI)	
		Model 1^b^	Model 2^c^
**Genetic Risk**			
Low	137/848468.6	1.00[Reference]	1.00[Reference]
Intermediate	516/2497938.8	1.29[1.07, 1.56]	1.29[1.07, 1.56]
High	227/864631.8	1.63[1.32, 2.02]	1.63[1.32, 2.02]
*P* value for trend		< 0.0001	< 0.0001
Per SD		1.20[1.13, 1.29]	1.21[1.13, 1.29]
**Healthy Lifestyle Category**			
Poor	108/310549.1	1.00[Reference]	1.00[Reference]
Intermediate	482/2097693.1	0.65[0.53, 0.80]	0.65[0.53, 0.80]
Optimal	290/1802797	0.49[0.39, 0.61]	0.49[0.39, 0.61]
*P* value for trend		< 0.0001	< 0.0001
Per SD		0.77[0.72, 0.83]	0.77[0.72, 0.83]

*Abbreviation*: *CI* Confidence Interval, *SD* standard deviation

^a^Model discrimination was similar between the main Cox models including the PRS (Harrell’s C index 0.541 (se 0.009), lifestyle categories (0.572 (se 0.009)), and the combined genetic and lifestyle categories (0.591 (se 0.01))

^b^ Model 1: Cox proportional hazards regression models were adjusted for age, sex, socioeconomic status (index of multiple deprivation, fifth), family history of cancer and first 10 principal components of ancestry

^c^Additionally, mutually adjusted for genetic risk and healthy lifestyle categories

### Associations of lifestyle with incident bladder cancer

We also observed a significant association between lifestyle score and a reduced bladder cancer risk (Fig. 1 & Table 2). Participants with an intermediate and optimal lifestyle had 35% and 51% reductions in risk of bladder cancer compared with those with poor lifestyle (HR 0.65, 95% CI 0.53–0.80; HR 0.49, 95% CI 0.39–0.61, respectively), and the estimated HR was similar after additional adjustment for PRS. Among individual lifestyle components, body weight, smoking and diet were significantly associated with the risk of bladder cancer, whereas physical activity was not associated with the risk (Table 3). And we also found that smoking status contributed most to the risk of incident bladder cancer.

**Table 3:** Associations between lifestyle components and risk of bladder cancer

	Cases/ Person-years	Hazard ratio (95% CI)	
		Model 1^a^	Model 2^b^
Body weight			
Poor	384/1511732	1.00[Reference]	1.00[Reference]
Intermediate	324/1540220	0.78[0.67, 0.90]	0.79[0.68, 0.92]
Optimal	172/1159088	0.74[0.62, 0.88]	0.74[0.62, 0.89]
Physically active			
Poor	117/507092	1.00[Reference]	1.00[Reference]
Intermediate	282/1432466	0.87[0.70, 1.08]	0.93[0.75, 1.16]
Optimal	481/2271481	0.88[0.72, 1.08]	0.99[0.80, 1.21]
Smoking			
Poor	162/411681	1.00[Reference]	1.00[Reference]
Intermediate	178/784923	0.52[0.42, 0.64]	0.52[0.42, 0.65]
Optimal	540/3014436	0.42[0.35, 0.50]	0.43[0.36, 0.52]
Diet			
Poor	343/1366028	1.00[Reference]	1.00[Reference]
Intermediate	211/965920	0.89[0.75, 1.06]	0.95[0.80, 1.13]
Optimal	326/1879091	0.73[0.63, 0.85]	0.81[0.69, 0.95]

*Abbreviation*: *CI* Confidence Interval, *HR* hazard ratio

^a^ Model 1: Cox proportional hazards regression models were adjusted for age, sex, socioeconomic status (index of multiple deprivation, fifth), family history of cancer and first 10 principal components of ancestry

^b^Additionally, mutually adjusted for four healthy lifestyle factors

### Joint effect of genetic and lifestyle factors on bladder cancer risk

**Fig. 2:**
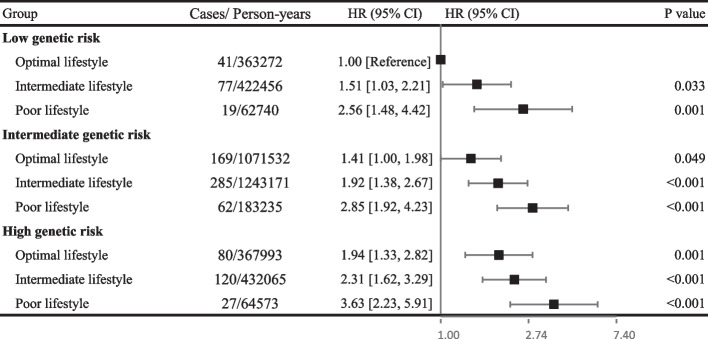
Risk of incident bladder cancer according to genetic and lifestyle risk. Abbreviation: HR, hazard ratio. Cox regression model was adjusted for age, sex, socioeconomic status (index of multiple deprivation, fifth), family history of cancer and first 10 principal components of ancestry

Figure 2 showed a combined effect of genetic variants and lifestyle factors on the risk of bladder cancer. The risk of bladder cancer increased as both genetic risk and poor lifestyle-related risk increased (Supporting Information Figure S2). Participants with a high genetic risk and a poor lifestyle had 3.6-fold elevated risk of bladder cancer compared with those with a low genetic risk and an optimal lifestyle (HR 3.63, 95% CI 2.23 -5.91). We did not find sufficient evidence of a significant multiplicative interaction or an additive interaction between genetic and lifestyle factors on bladder cancer (*P* = 0.837, Supporting Information Table S6). Additionally, 19.8% of new-onset bladder cancer events during follow-up would be prevented if all individuals adopted an optimal lifestyle (Supporting Information Table S7).

### Benefits of adopting an optimal lifestyle with bladder cancer

In further analyses stratified by PRS categories with a poor lifestyle as the reference group, we found that an optimal lifestyle was associated with a lower risk of bladder cancer, regardless of PRS (Table 4). Within each genetic risk stratum, those with an intermediate or optimal lifestyle had a nearly 50% reduction in risk of bladder cancer as compared to participants with a poor lifestyle. For example, among participants with high PRS, participants with intermediate or optimal lifestyle had 38% and 49% lower risk of bladder cancer compared with those with poor lifestyle (HR 0.62, 95% CI 0.41–0.94; HR 0.51, 95% CI 0.33–0.80, respectively). Each 18 participants with high genetic risk who converted their lifestyle from poor to optimal may prevent 1 bladder cancer case in 10 years.

**Table 4:** Associations lifestyle score and risk of bladder cancer stratified by PRS category

Group	Cases/ Person-years	HR (95% CI)	P trend value	Risk difference over 10 year (‰)	The number needed to treat to benefit
**Low genetic risk**			0.001		
Poor lifestyle	19/ 62741	1.00[Reference]		1.00[Reference]	
Intermediate lifestyle	77/422456	0.61[0.36, 1.00]		26.05[0.02, 120.92]	38
Optimal lifestyle	41/363272	0.41[0.23, 0.70]		89.72[14.22, 260.41]	11
**Intermediate genetic risk**			< 0.001		
Poor lifestyle	62/183235	1.00[Reference]		1.00[Reference]	
Intermediate lifestyle	285/1243172	0.68[0.51, 0.89]		17.51[2.94, 51.57]	57
Optimal lifestyle	169/1071532	0.49[0.37, 0.66]		58.19[20.03, 118.48]	17
**High genetic risk**			0.008		
Poor lifestyle	27/64573	1.00[Reference]		1.00[Reference]	
Intermediate lifestyle	120/ 432065	0.62[0.41, 0.94]		29.33[1.63, 101.68]	34
Optimal lifestyle	80/367993	0.51[0.33, 0.80]		57.04[8.32, 159.89]	18

Cox proportional hazards regression models were adjusted for age, sex, socioeconomic status (index of multiple deprivation, fifth), family history of cancer and first 10 principal components of ancestry

*Abbreviation CI* Confidence Interval, *HR* hazard ratio

The observed associations of PRS and lifestyle with bladder cancer were consistent in men and women, although some observed associations were not significant and unreliable due to the relatively low number of cases (interaction *P* > 0.10, Supporting Information Figure S3-S4 &Table S8-10). And the joint effect of genetic and lifestyle factors on bladder cancer risk was not modified by smoking and diabetes (Supporting Information Table S11-12). These results remain essentially unchanged in a series of sensitivity analyses by excluding the incident bladder cancer identified during the first 2 years of follow-up, restricting the analysis to participants of white British descent, in the competing risk analysis using Fine-Gray subdistribution hazard model, or using a weighted lifestyle score (Supporting Information Table S13-15). In addition, the pattern of associations based on the healthy lifestyle scores without smoking status were similar (Supporting Information Figure S5). We also conducted an additional analysis to evaluate the joint effect of genetic and lifestyle factors on the early- and late-onset of bladder cancer risk, and found that the joint effect of genetic and lifestyle factors was more evident in the risk of bladder cancer diagnosed in adults < 60 years of age compared to that diagnosed in adults > 65 years (Supporting Information table S16).

## Discussion

In the present prospective population-based cohort study of 375 998 participants, we found that genetic risk and lifestyle factors were independently associated with risk of bladder cancer. Participants at high genetic risk had a 65% increased risk of bladder cancer, whereas adherence to an optimal lifestyle was associated with an approximately 50% reduction in the risk of bladder cancer across all genetic risk strata. Participants with a high genetic risk and a poor lifestyle had a more than threefold elevated risk of bladder cancer compared with those with a low genetic risk and an optimal lifestyle. Furthermore, we found that an intermediate lifestyle was also associated with a 40% lower risk of bladder cancer regardless of genetic risk.

Our findings suggested that a polygenic risk score was associated with bladder cancer risk independently of lifestyle-related factors and other putative risk factors, which was consistent with those of previous studies for other cancers and other diseases [18, 19, 21, 32–35]. For example, a recent analysis showed that individuals in the highest 5% of the PRS had a nearly 50% higher risk of cancer of the bladder, lung or kidney, and a more than threefold increased risk of cancer of the prostate, breast, pancreas, colorectal, or ovary as compared to those at an average risk [7]. However, this analysis did not account for the influence of lifestyle factors. Another pan-cancer analysis also showed that integrating PRS can efficiently improve prediction accuracy for most cancers including bladder cancer, but the study only evaluated overall neoplasm risk (included a borderline, in situ, or malignant primary cancers), and genetic risk might differ by pathological classification [8]. These findings, including the results of our study, supported that familial or genetic predisposition could increase bladder cancer risk, and PRS may be used to identify high-risk individuals for bladder cancer.

Our study also indicated a combined healthy lifestyle was associated with a reduced risk of bladder cancer within and across genetic risk groups, which was in line with those of previous studies of site-specific cancers like breast, stomach and colorectal cancers [19–21]. Previous meta-analysis has shown that fruit and vegetable intake, tea intake, and physical activity were protective factors for bladder cancer, while smoking and obesity were risk factors [11]. For example, evidence from the BLEND study demonstrated that the Mediterranean diet might reduce the risk of bladder cancer, while the Western dietary pattern might increase the risk [13, 16]. However, the current evidence of the association between the combined lifestyle factors and bladder cancer risk was scarce. A meta-analysis including two studies of bladder cancer reported that adherence to a healthy lifestyle was associated with a 17% lower risk of bladder cancer [22]. However, further inspection of the two original studies found that two publications did not report the estimated effects of combined lifestyle factors on bladder cancer risk [36, 37].

Our work further supported that a healthy lifestyle could powerfully reduce bladder cancer risk regardless of the individual’s genetic risk profile. In line with our findings, a case–control study found that high intake of red meat was associated with an increased bladder cancer risk, and the association was not modified by genetic variants in the metabolic pathways of HCAs [38]. On the contrary, several studies have suggested that lifestyle factors may interact with genetic variants to modify the risk of developing bladder cancer [38–42]. For example, a case–control study conducted in the US showed that the association between cruciferous vegetable intake and bladder cancer risk might be modified by the GSTM1 genotype, and the protective effect of cruciferous vegetables was observed only in subjects carrying the NAT2-slow genotype [39]. Another study demonstrated an interaction between diet quality and the variant rs8102137, and the reduced risk of adherence to the AHEI-2010 long-chain fats guideline was evident among subjects with a protective rs8102137 allele (genotype TT), but not among those carrying the at-risk CT or CC genotypes [41]. However, a limited study has examined the potential interactions of aggregated genetic risk and overall healthy lifestyle in relation to bladder cancer risk. In the present study, we constructed a PRS and healthy lifestyle score, and observed insufficient evidence of an interaction involving the genetic susceptibility, overall lifestyle score and bladder cancer risk. The reduced risk of bladder cancer associated with a healthy lifestyle in present study was similar across all stratums of genetic risk, suggesting the benefit for entire populations of adopting a healthy lifestyle, regardless of genetic risk.

We also found that participants with a high genetic risk and a poor lifestyle had the highest risk of bladder cancer, and the detrimental effect of genetic risk could be offset by adopting a healthy lifestyle. Similar patterns were observed in the previous studies [38, 43]. For example, a case-study revealed that individuals carrying six or more unfavorable genotypes in the metabolic pathways of HCAs (such as GSTA1, GSTM5, NAT2, and GSTP1) and with the highest intake of red meat had the greatest risk of bladder cancer (OR 5.09; 95% CI: 2.89–8.96), but the interaction was not significant [38]. However, we did not detect an additive interaction between genetic and lifestyle factors in relation to bladder cancer, which has been reported for overall cancer, colorectal cancer, and breast cancer previously [18, 20, 44]. Therefore, we were unable to infer whether the joint effects of genetic and lifestyle factors were greater than the sum of the individual effects of lifestyle and PRS. Additionally, our results also suggested that adopting even a few of these healthy behaviors can offer benefits, which aligns recommendations in guidelines, like “some physical activity is better than none”, or “smoking cessation is beneficial at any age” [45, 46]. Therefore, effective policies and behavioral interventions to encourage individuals to adopt a healthy lifestyle across the entire population, particularly for those at high genetic risk, are necessary to mitigate bladder cancer risk.

### Strengths and limitations

The main strength was that this study was based on a well-designed prospective cohort of over 370 000 participants with genetic data and 880 events during up to 14.8 years of follow-up, providing sufficient statistical power to explore the effect of the combination of genetic risk and lifestyle on the bladder cancer in detail. In addition, several robust sensitivity analyses further strengthened the validity of our findings.

Our study also has several limitations. Firstly, some of lifestyle factors were self-reported, which may introduce recall bias and misclassification errors in assessing lifestyle risk levels. However, misclassification errors seemed more likely to drive associations towards the null. Secondly, the lifestyle factors were measured only once at baseline, so we were unable to evaluate the long-term effects of lifestyle behaviors. Thirdly, in the present study, the HLS did not include all components of the WCRF/AICR cancer prevention recommendations because information on the consumption of fast foods and sugar-sweetened drinks was not collected in the UK Biobank at baseline. Fourthly, given that those susceptibility SNPs were identified among people of European descent and our study population restricted to volunteers of European descent, the generalizability of our finding to populations with distinct ancestry should be further examined in future studies. Lastly, due to the observational nature of the study, we cannot establish a causal association between the lifestyle behaviors and bladder cancer risk. Although we have controlled for known potential sources of bias in our analyses, the possibility of unmeasured confounding (e.g., health literacy, socioeconomic status) and reverse bias cannot be fully excluded. However, the findings remained robust after excluding bladder cancer cases occurred within the first 2 years, suggesting that the reverse bias would be likely to be minimal.

## Conclusion

In summary, our study suggested that adhering to an intermediate or optimal lifestyle can reduce the risk of bladder cancer across all genetic risk strata of the population, even in those at a high genetic risk of bladder cancer. For all populations, adopting an intermediate lifestyle is more beneficial than a poor one, and adhering to an optimal lifestyle is the ideal effective strategy for bladder cancer prevention, especially among individuals with a high genetic risk.

### Supplementary Information

**Additional file 1.** The file contains additional analysis. **Table S1.** Definitions of lifestyle factors. **Table S2.** Singlenucleotide polymorphisms used to build the genetic risk score for bladder cancer. **Table S3. **Associations between the polygenic risk score and lifestyle factors. **Table S4.** Risk of bladder cancer according to different risk levels of polygenic risk score. **Table S5.** Hazard ratio (95% CI) of bladder cancer according to genetic risk category in sensitivity analyses. **Table S6.** RERI, AP and SI for additive interaction between genetic and lifestyle categories. **Table S7.** Population-attributable fraction per behavioral lifestyle group. **Table S8.** Association of genetic and lifestyle factors with bladder cancer risk stratified by gender. **Table S9.** Associations of lifestyle categories and risk of bladder cancer stratified by PRS category and gender. **Table S10.** Risk of incident bladder cancer according to genetic and lifestyle category stratified by gender. **Table 11.** Risk of incident bladder cancer according to genetic and lifestyle risk stratified by smoking status. **Table S12.** Risk of incident bladder cancer according to genetic and lifestyle risk stratified by smoking status. **Table S13.** Hazard ratio (95% CI) of bladder cancer according to lifestyle category in sensitivity analyses. **Table S14.** Associations of lifestyle score and risk of bladder cancer stratified by PRS category in sensitivity analyses. **Table S15.** Risk of incident bladder cancer according to genetic and lifestyle risk in sensitivity analyses. **Table S16.** The joint effect of genetic and lifestyle factors on early-and late-onset of bladder cancer risk. **Figure S1.** Flowchart for the selection of the study population from UK Biobank study. **Figure S2.** Risk of incident bladder cancer according to genetic risk and lifestyle factors. **Figure S3.** Risk of incident bladder cancer according to genetic risk stratified by sex. **Figure S4.** Risk of incident bladder cancer according to lifestyle profile stratified by sex. **Figure S5.** Risk of incident bladder cancer according to genetic and lifestyle risk (without smoking status).

## Data Availability

The data that support the findings of this study are available from UK Biobank (application number 51671, approved August 2019) but restrictions apply to the availability of these data, which were used under license for the current study, and so are not publicly available. Data are however available from the authors upon reasonable request and with permission of UK Biobank. The UK Biobank is an open access resource and researchers can apply to use the UK Biobank dataset by registering and applying at website (https://www.ukbiobank.ac.uk/). Data sets used for the analysis will be made available under reasonable requests. All participants provided written informed consent prior to data collection.

## Abbreviations

BMI: Body mass index
CI: Confidence interval
GWAS: Genome-wide association studies
HLS: Healthy lifestyle score
HR: Hazard ratio
ICD: International Classification of Diseases
IPAQ: International Physical Activity Questionnaire
PRS: Polygenic risk score
SNP: Single nucleotide polymorphisms
UK: United Kingdom
WC: Waist circumference
WCRF/AICR: World Cancer Research Fund/American Institute of Cancer Research
