# Supplementary material for: Genetic factors, adherence to healthy lifestyle behaviors, and risk of bladder cancer
Source: BMC Cancer. 2023 Oct 12;23:965. doi: 10.1186/s12885-023-11455-4 (PMC10568887; doi:10.1186/s12885-023-11455-4)
Supplement: Supplementary file 1 — Additional file 1. The file contains additional analysis. Table S1. Definitions of lifestyle factors. Table S2. Singlenucleotide polymorphisms used to build the genetic risk score for bladder cancer. Table S3. Associations between the polygenic risk score and lifestyle factors. Table S4. Risk of bladder cancer according to different risk levels of polygenic risk score. Table S5. Hazard ratio (95% CI) of bladder cancer according to genetic risk category in sensitivity analyses. Table S6. RERI, AP and SI for additive interaction between genetic and lifestyle categories. Table S7. Population-attributable fraction per behavioral lifestyle group. Table S8. Association of genetic and lifestyle factors with bladder cancer risk stratified by gender. Table S9. Associations of lifestyle categories and risk of bladder cancer stratified by PRS category and gender. Table S10. Risk of incident bladder cancer according to genetic and lifestyle category stratified by gender. Table 11. Risk of incident bladder cancer according to genetic and lifestyle risk stratified by smoking status. Table S12. Risk of incident bladder cancer according to genetic and lifestyle risk stratified by smoking status. Table S13. Hazard ratio (95% CI) of bladder cancer according to lifestyle category in sensitivity analyses. Table S14. Associations of lifestyle score and risk of bladder cancer stratified by PRS category in sensitivity analyses. Table S15. Risk of incident bladder cancer according to genetic and lifestyle risk in sensitivity analyses. Table S16. The joint effect of genetic and lifestyle factors on early-and late-onset of bladder cancer risk. Figure S1. Flowchart for the selection of the study population from UK Biobank study. Figure S2. Risk of incident bladder cancer according to genetic risk and lifestyle factors. Figure S3. Risk of incident bladder cancer according to genetic risk stratified by sex. Figure S4. Risk of incident bladder cancer according to lifestyle profile stratified by [file 12885_2023_11455_MOESM1_ESM.docx]

**SUPPLEMENTARY** **MATERIALS**

**Genetic Factors, Adherence to Healthy Lifestyle Behaviors, and Risk of Bladder Cancer**

**Table of Contents**

| **Page** |  |
| --- | --- |
| **3-4** | **Table S1.** Definitions of lifestyle factors |
| **5** | **Table S2.** Single-nucleotide polymorphisms used to build the genetic risk score for bladder cancer |
| **6** | **Table S3.** Associations between the polygenic risk score and lifestyle factors |
| **7** | **Table S4.** Risk of bladder cancer according to different risk levels of polygenic risk score |
| **8** | **Table S5.** Hazard ratio (95% CI) of bladder cancer according to genetic risk category in sensitivity analyses |
| **9** | **Table S6.** RERI, AP and SI for additive interaction between genetic and lifestyle categories |
| **10** | **Table S7.** Population-attributable fraction per behavioral lifestyle group |
| **11** | **Table S8.** Association of genetic and lifestyle factors with bladder cancer risk stratified by gender |
| **12** | **Table S9**. Associations of lifestyle categories and risk of bladder cancer stratified by PRS category and gender |
| **13** | **Table S10.** Risk of incident bladder cancer according to genetic and lifestyle category stratified by gender |
| **14** | **Table S11.** Risk of incident bladder cancer according to genetic and lifestyle risk stratified by smoking status |
| **15** | **Table S12.** Risk of incident bladder cancer according to genetic and lifestyle risk stratified by diabetes |
| **16** | **Table S13.** Hazard ratio (95% CI) of bladder cancer according to lifestyle category in sensitivity analyses |
| **17** | **Table S14**. Associations of lifestyle score and risk of bladder cancer stratified by PRS category in sensitivity analyses |
| **18** | **Table S15.** Risk of incident bladder cancer according to genetic and lifestyle risk in sensitivity analyses |
| **19** | **Table S16.** The joint effect of genetic and lifestyle factors on early-and late-onset of bladder cancer risk |
| **20** | **Figure S1.** Flowchart for the selection of the study population from UK Biobank study |
| **21** | **Figure S2.** Risk of incident bladder cancer according to genetic risk and lifestyle factors |
| **22** | **Figure S3**. Risk of incident bladder cancer according to genetic risk stratified by sex |
| **23** | **Figure S4.** Risk of incident bladder cancer according to lifestyle profile stratified by sex |
| **24** | **Figure S5.** Risk of incident bladder cancer according to genetic and lifestyle risk (without smoking status) |

|  | Table S1. Definitions of lifestyle factors | | | | | |
| --- | --- | --- | --- | --- | --- | --- |
| Healthy lifestyle factor | | **Self-reported UK Biobank field code** | **Source and definition** | | **Score** | **Categories** |
| Body weight* | | BMI: 21001 | UK Biobank Physical measures at baseline; |  | |  |
|  |  |  | 18.5-24.9 kg/m^2^ | 1 | 2 | Optimal |
|  |  |  | 25.0-29.9 kg/m^2^ | 0.5 |  |  |
|  |  |  | ≥30 kg/m^2^ | 0 | >0&<2 | Intermediate |
|  |  | Waist circumference (WC): 48 | WC <94 cm in men; WC <80 cm in women | 1 |  |  |
|  |  |  | 94≤WC<102 in men and 80 ≤ WC<88 in women | 0.5 | 0 | Poor |
|  |  |  | WC ≥102 cm in men and WC ≥88cm | 0 |  |  |
| Smoking | | 20116, 2897 | UK Biobank Touchscreen questionnaire at baseline; | |  |  |
|  |  |  | Never smoked or quit 10 years ago | | 2 | Optimal |
|  |  |  | Previous smokers | | 1 | Intermediate |
|  |  |  | Current smokers | | 0 | Poor |
| Physical activity | | 884, 894, 904, 914 | UK Biobank Touchscreen questionnaire at baseline; | |  |  |
|  |  |  | ≥150 min/week ≥150 minutes per week moderate or ≥75 minutes per week vigorous or an equivalent combination | | 2 | Optimal |
|  |  |  | 1– 149 min/week moderate or 1–74 min/week vigorous or 1–149 min/week mixed activity | | 1 | Intermediate |
|  |  |  | Performing any moderate or vigorous activity | | 0 | Poor |
| Diet | | 1309, 1319, 1289, 1299,1438, 1448, 1458, 1468,1349,1369, 1379, 1389, 1488 | UK Biobank Food Frequency Questionnaire at baseline; | |  |  |
| Total fruit and vegetable intake | | 1309, 1319, 1289, 1299 | ≥5servings/day | | 2 | Diet score was categorized in tertiles: “Poor” (lowest tertile of diet score), “intermediate” (second tertile), “Optimal” (highest tertile). |
|  |  |  | ≥3-<5 servings/day | | 1 |  |
|  |  |  | <3 serving/day | | 0 |  |
| Whole grains intake | | 1438, 1448, 1458, 1468 | ≥3servings/day | | 2 |  |
|  |  |  | >1-3 servings/day | | 1 |  |
|  |  |  | ≤1 serving/day | | 0 |  |
| Red meat and processed meat | | 1349,1369, 1379, 1389, | Red meat≤ 1 times/week & processed meat ≤ 1 times/week | | 2 |  |
|  |  |  | Red meat≤ 1 times/week & processed meat 2-4 times/week | | 1 |  |
|  |  |  | Red meat> 1 times/week or processed meat ≤ 1.7 times/week | | 0 |  |
| Tea intake | | 1488 | 4 cups/day | | 1 |  |
|  |  |  | 2-3 cups/day | | 1 |  |
|  |  |  | 0-1 cups/day | | 0 |  |

Note: ^*^The score for body weight was the sum of BMI points and waist circumference points. When data are available for both BMI and waist circumference, the sum of the two will be used to score the body weight.

A weighted healthy lifestyle score was computed based on the weighted β coefficients of each lifestyle factor with bladder cancer risk in Cox model with adjustment for other lifestyle factors and confounders. The original lifestyle variables were multiplied by the β coefficients, summed, divided by the sum of the β coefficients, multiplied by 100. The weighted standardized lifestyle score was categorized into 3 ordered categories on tertiles (optimal, intermediate, poor).

| **Table S2.** Single-nucleotide polymorphisms used to build the genetic risk score for bladder cancer | | | | | | | | | |
| --- | --- | --- | --- | --- | --- | --- | --- | --- | --- |
| Reference | Gene | Variant | Chr | Position | RAF | RA | Ref | beta | P value |
| Figueroa; Hum Mol Genet (2014) | MYNN | rs10936599 | 3 | 169492101 | 0.75 | C | T | 0.162518929 | 4.53E-09 |
| Figueroa; Hum Mol Genet (2014) | TP63/LEPREL1 | rs710521 | 3 | 189645933 | 0.72 | T | C | 0.131028262 | 1.92E-11 |
| Figueroa; Hum Mol Genet (2014) | TMEM129-TACC3-FGFR3 | rs798766 | 4 | 1734239 | 0.19 | T | C | 0.198850859 | 7.06E-25 |
| Figueroa; Hum Mol Genet (2014) | TERT/CLPTM1L | rs401681 | 5 | 1322087 | 0.56 | C | T | 0.113328685 | 4.27E-11 |
| Rothman; Nat Genet (2010) | NAT2 | rs1495741 | 8 | 18272881 | 0.78 | A | G | 0.139761942 | 4.20E-11 |
| Figueroa; Hum Mol Genet (2014) | c-MYC | rs9642880 | 8 | 128718068 | 0.45 | T | G | 0.21511138 | 3.55E-38 |
| Figueroa; Hum Mol Genet (2014) | PSCA | rs2294008 | 8 | 143761931 | 0.44 | T | C | 0.122217633 | 2.75E-15 |
| Figueroa; Hum Mol Genet (2014) | LSP1 | rs907611 | 11 | 1874072 | 0.32 | A | G | 0.139761942 | 4.11E-08 |
| Figueroa; Hum Mol Genet (2016) | MCF2L | rs4907479 | 13 | 113659706 | 0.27 | A | C | 0.122217633 | 3.30E-10 |
| Rafnar; Hum Mol Genet (2011) | SLC14A1 | rs17674580 | 18 | 43309911 | 0.36 | T | C | 0.157003749 | 7.60E-11 |
| Rafnar; Hum Mol Genet (2011) | SLC14A1 | rs1058396 | 18 | 43319519 | 0.50 | G | A | 0.131028262 | 2.90E-09 |
| Rothman; Nat Genet (2010) | CCNE1 | rs8102137 | 19 | 30296853 | 0.33 | C | T | 0.122217633 | 1.70E-11 |
| Rafnar; Hum Mol Genet (2014) | JAG1 | rs62185668 | 20 | 10977631 | 0.29 | G | A | 0.173953307 | 1.50E-11 |
| Figueroa; Hum Mol Genet (2016) |  | rs6104690 | 20 | 10984105 | 0.33 | C | T | 0.127833372 | 2.19E-11 |
| Rafnar; Hum Mol Genet (2014) |  | rs4813953 | 20 | 10991138 | 0.38 | T | C | 0.148420005 | 2.10E-10 |
| Rothman; Nat Genet (2010) | CBX6, APOBEC3A | rs1014971 | 22 | 39332623 | 0.64 | T | C | 0.131028262 | 8.40E-12 |

| **Table S3**. Associations between the polygenic risk score and lifestyle factors | | |
| --- | --- | --- |
|  | **OR (95% CI) ^a^** | ***P* value** |
| Healthy weight |  |  |
| Poor | 1.00[Reference] |  |
| Intermediate | 1.00[0.99, 1.01] | 0.642 |
| Optimal | 1.00[0.99, 1.02] | 0.970 |
| Smoking |  |  |
| Poor | 1.00[Reference] |  |
| Intermediate | 0.99[0.97, 1.00] | 0.052 |
| Optimal | 0.99[0.98, 1.00] | 0.039 |
| Physically active |  |  |
| Poor | 1.00[Reference] |  |
| Intermediate | 1.00[0.99, 1.01] | 0.768 |
| Optimal | 1.00[0.99, 1.02] | 0.892 |
| Diet |  |  |
| Poor | 1.00[Reference] |  |
| Intermediate | 0.99[0.98, 1.00] | 0.183 |
| Optimal | 0.99[0.98, 1.00] | 0.055 |
| Healthy Lifestyle Category |  |  |
| Poor | 1.00[Reference] |  |
| Intermediate | 1.00[0.98, 1.01] | 0.482 |
| Optimal | 0.99[0.98, 1.00] | 0.130 |

^a^ Adjusted for age, sex, socioeconomic status (index of multiple deprivation, fifth), and first 10 principal components of ancestry;
Abbreviations: CI, confidence interval; OR, odds ratio; PRS, polygenic risk score.

| **Table S4.** Risk of bladder cancer according to different risk levels of polygenic risk score | | | |
| --- | --- | --- | --- |
|  | Cases/ Person-years | HR [95% CI] | P value |
| Tertiles of PRS |  |  |  |
| Tertile 1 | 238/1442776 | 1.00[Reference] |  |
| Tertile 2 | 300/1420912 | 1.28[1.08, 1.52] | 0.004 |
| Tertile 3 | 342/1347351 | 1.54[1.30, 1.82] | <0.0001 |
| P value for trend |  | <0.0001 |  |
| Quartiles of PRS |  |  |  |
| Q1 | 173/1080495 | 1.00[Reference] |  |
| Q2 | 228/1067517 | 1.32[1.06, 1.63] | 0.013 |
| Q3 | 239/1128739 | 1.33[1.07, 1.64] | 0.009 |
| Q4 | 240/934288 | 1.65[1.34, 2.04] | <0.0001 |
| P value for trend |  | <0.001 |  |
| Quartiles of PRS |  |  |  |
| Low (Q1) | 173/1080495 | 1.00[Reference] |  |
| Intermediate (Q2-Q3) | 4467/2196256 | 1.32[1.09, 1.60] | 0.004 |
| High (Q4) | 240/934288 | 1.65[1.34, 2.04] | <0.0001 |
| P value for trend |  | <0.0001 |  |

Estimated HRs were based on the adjusted Cox proportional hazards regression models, with adjustment for age, sex, socioeconomic status (index of multiple deprivation, fifth), family history of cancer and first 10 principal components of ancestry and healthy lifestyle categories.

Abbreviation: HR, hazard ratio; CI, Confidence Interval.

| **Table S5.** Hazard ratio (95% CI ) of bladder cancer according to genetic risk category in sensitivity analyses | | | | |
| --- | --- | --- | --- | --- |
|  | Cases/ Person-years | Hazard ratio (95% CI) | P value | P trend value |
| **Limiting the participants in white British descent** |  |  |  | <0.0001 |
| Low genetic risk | 124/766695 | 1.00[Reference] |  |  |
| Intermediate genetic risk | 475/2254754 | 1.32[1.08, 1.61] | 0.006 |  |
| High genetic risk | 202/782188 | 1.61[1.29, 2.01] | <0.0001 |  |
| Per SD |  | 1.19[1.11, 1.28] | <0.0001 |  |
| **Competing Risk Analysis using Fine-Gray subdistribution hazard model** |  |  |  | <0.0001 |
| Low genetic risk | 137/848469 | 1.00[Reference] |  |  |
| Intermediate genetic risk | 516/2497939 | 1.27[1.03, 1.56] | 0.023 |  |
| High genetic risk | 227/864632 | 1.68[1.34, 2.12] | <0.0001 |  |
| Per SD |  | 1.22[1.14, 1.32] | <0.0001 |  |

Estimated HRs were based on the adjusted Cox proportional hazards regression models, with adjustment for age, sex, socioeconomic status (index of multiple deprivation, fifth), family history of cancer and first 10 principal components of ancestry and healthy lifestyle categories.

Abbreviation: HR, hazard ratio; CI, Confidence Interval.

| **Table S6.** RERI, AP and SI for additive interaction between genetic and lifestyle categories | | | | | | |  |
| --- | --- | --- | --- | --- | --- | --- | --- |
| Lifestyle category | Genetic risk | | | | | |  |
|  | Intermediate | | |  | High | | |
|  | RERI [95%CI] | AP [95%CI] | SI [95%CI] |  | RERI [95%CI] | AP [95%CI] | SI [95%CI] |
| Intermediate | 0.01[-0.77, 0.43] | 0 [-0.31, 0.28] | 1.00 [0.55, 1.82] |  | -0.14 [-1.15, 0.53] | -0.06 [-0.44, 0.23] | 0.90 [0.53, 1.53] |
| Poor | -0.12 [-1.98, 1.14] | -0.04 [-0.69, 0.35] | 0.94 [0.46, 1.94] |  | 0.13 [-2.00, 2.17] | 0.04 [-0.67, 0.40] | 1.05 [0.51, 2.18] |

Adjusted for age, sex, socioeconomic status (index of multiple deprivation, fifth), family history of cancer and first 10 principal components of ancestry.

Abbreviations: RERI, relative excess risk due to interaction; AP, attributable proportion due to interaction; CI, confidence interval; SI, synergy index

| **Table S7**. Population-attributable fraction per behavioral lifestyle group | |
| --- | --- |
|  | PAF (%) [ 95% CI] |
| Non-optimal to optimal lifestyle | 19.8 [11.9, 26.9] |
| Intermediate to optimal lifestyle | 14.4 [7.19, 21.5] |
| Poor to optimal lifestyle | 7.45 [4.68, 10.7] |

Abbreviations: PAF, Population Attributable Fraction; CI, Confidence Interval

| **Table S8**. Association of genetic and lifestyle factors with bladder cancer risk stratified by gender | | | | | |
| --- | --- | --- | --- | --- | --- |
|  | **Male** | | **Female** | | P for interaction |
|  | Cases/ Person-years | Hazard ratio (95% CI) | Cases/ Person-years | Hazard ratio (95% CI) |  |
| Genetic Risk |  |  |  |  | 0.624 |
| Low | 137/502779 | 1.00[Reference] | 36/57771 | 1.00[Reference] |  |
| Intermediate | 363/1019362 | 1.31[1.08, 1.60] | 104/1176893 | 1.42[0.97, 2.07] |  |
| High | 194/433070 | 1.64[1.32, 2.05] | 46/501218 | 1.47[0.95, 2.28] |  |
| P value for trend |  | <0.001 |  | 0.084 |  |
| Per SD |  | 1.21[1.12, 1.30] |  | 1.19[1.03, 1.37] |  |
| Healthy Lifestyle Category |  |  |  |  |  |
| Poor | 85/164473 | 1.00[Reference] | 23/146075 | 1.00[Reference] | 0.417 |
| Intermediate | 392/1031393 | 0.69[0.54, 0.87] | 90/1066300 | 0.51[0.32, 0.81] |  |
| Optimal | 217/759344 | 0.50[0.39, 0.64] | 73/1043452 | 0.44[0.27, 0.70] |  |
| P value for trend |  | <0.001 |  | 0.005 |  |
| Per SD |  | 0.78[0.73, 0.84] | 0.77[0.72, 0.83] | 0.74[0.64, 0.85] |  |

Estimated HRs were based on the adjusted Cox proportional hazards regression models, with adjustment for age, socioeconomic status (index of multiple deprivation, fifth), family history of cancer and first 10 principal components of ancestry, genetic risk, and healthy lifestyle categories.

Abbreviation: CI, Confidence Interval; SD, standard deviation.

| **Table S9.** Associations lifestyle categories and risk of bladder cancer stratified by PRS category and gender | | | | | | | | | | | |
| --- | --- | --- | --- | --- | --- | --- | --- | --- | --- | --- | --- |
|  | **Low genetic risk** | | |  | **Intermediate genetic risk** | | |  | **High genetic risk** | | |
|  | Cases/ Person-years | Hazard ratio  (95% CI) | P trend value |  | Cases/ Person-years | Hazard ratio  (95% CI) | P trend value |  | Cases/ Person-years | Hazard ratio  (95% CI) | P trend value |
| **Men** |  |  | 0.31 |  |  |  | 0.042 |  |  |  | 0.02 |
| Poor lifestyle | 15/41890 | 1.00[Reference] |  |  | 44/86513 | 1.00[Reference] |  |  | 26/36070 | 1.00[Reference] |  |
| Intermediate lifestyle | 75/264450 | 0.75[0.43, 1.30] |  |  | 208/536874 | 0.71[0.51, 0.99] |  |  | 119/230068 | 0.60[0.39, 0.92] |  |
| Optimal lifestyle | 47/196438 | 0.62[0.34, 1.11] |  |  | 111/395974 | 0.49[0.34, 0.70] |  |  | 59/166931 | 0.43[0.27, 0.68] |  |
| **Women** |  |  | 0.02 |  |  |  | 0.048 |  |  |  | 0.71 |
| Poor lifestyle | 7/37604 | 1.00[Reference] |  |  | 13/74713 | 1.00[Reference] |  |  | 3/33758 | 1.00[Reference] |  |
| Intermediate lifestyle | 18/272846 | 0.35[0.15, 0.85] |  |  | 53/556986 | 0.54[0.29, 0.99] |  |  | 19/236468 | 0.79[0.23, 2.69] |  |
| Optimal lifestyle | 11/267267 | 0.23[0.09, 0.60] |  |  | 38/545194 | 0.41[0.22, 0.78] |  |  | 24/230991 | 1.01[0.30, 3.40] |  |

Estimated HRs were based on the adjusted Cox proportional hazards regression models, with adjustment for age, socioeconomic status (index of multiple deprivation, fifth), family history of cancer and first 10 principal components of ancestry.

Abbreviation: CI, Confidence Interval.

| **Table S10.** Risk of incident bladder cancer according to genetic and lifestyle category stratified by gender | | | | |  |
| --- | --- | --- | --- | --- | --- |
|  | **Men** | | **Women** | | P for interaction |
|  | Cases/ Person-years | Hazard ratio (95% CI) | Cases/ Person-years | Hazard ratio (95% CI) |  |
| **Low genetic risk** |  |  |  |  | 0.548 |
| Optimal lifestyle | 47/196438 | 1.00[Reference] | 7/208813 | 1.00[Reference] |  |
| Intermediate lifestyle | 75/264450 | 1.22[0.85, 1.76] | 11/213650 | 1.57[0.74, 3.32] |  |
| Poor lifestyle | 15/41890 | 1.65[0.92, 2.95] | 5/29514 | 4.52[1.74, 11.69] |  |
| **Intermediate genetic risk** |  |  |  |  |  |
| Optimal lifestyle | 111/395974 | 1.16[0.83, 1.64] | 32/620530 | 1.69[0.87, 3.31] |  |
| Intermediate lifestyle | 208/536874 | 1.69[1.23, 2.32] | 50/632941 | 2.25[1.17, 4.30] |  |
| Poor lifestyle | 44/86513 | 2.36[1.56, 3.57] | 12/85127 | 4.31[1.92, 9.66] |  |
| **High genetic risk** |  |  |  |  |  |
| Optimal lifestyle | 59/166931 | 1.47[1.00, 2.16] | 19/212834 | 2.53[1.24, 5.16] |  |
| Intermediate lifestyle | 119/230068 | 2.04[1.45, 2.88] | 16/218264 | 1.90[0.90, 3.99] |  |
| Poor lifestyle | 26/36070 | 3.36[2.08, 5.43] | 3/31156 | 2.23[0.62, 8.02] |  |

Estimated HRs were based on the adjusted Cox proportional hazards regression models, with adjustment for age, socioeconomic status (index of multiple deprivation, fifth), family history of cancer and first 10 principal components of ancestry.

Abbreviation: CI, Confidence Interval.

| **Table S11**. Risk of incident bladder cancer according to genetic and lifestyle risk stratified by smoking status | | | | | | | |
| --- | --- | --- | --- | --- | --- | --- | --- |
|  | Current smokers | | Previous smokers | | Never smokers | | P for interaction |
|  | Cases/  Person-years | Hazard ratio  (95% CI) | Cases/  Person-years | Hazard ratio  (95% CI) | Cases/  Person-years | Hazard ratio  (95% CI) |  |
| **Low genetic risk** |  |  |  |  |  |  | 0.643 |
| Optimal lifestyle | 7/3147 | 1.00[Reference] | 11/797533 | 1.00[Reference] | 33/317721 | 1.00[Reference] |  |
| Intermediate lifestyle | 15/37326 | 1.64[0.67, 4.02] | 17/619699 | 1.82[0.85, 3.90] | 35/22972 | 1.38[0.86, 2.23] |  |
| Poor lifestyle | 5/131299 | 1.61[0.51, 5.06] | 7/18126 | 2.46[0.95, 6.35] | 7/59250 | 1.11[0.49, 2.51] |  |
| **Intermediate genetic risk** |  |  |  |  |  |  |  |
| Optimal lifestyle | 37/93859 | 1.74[0.78, 3.92] | 34/22816 | 1.11[0.56, 2.20] | 149/9413877 | 1.54[1.06, 2.24] |  |
| Intermediate lifestyle | 48/11158 | 1.82[0.82, 4.02] | 46/1830955 | 1.68[0.87, 3.25] | 128/6767899 | 1.73[1.18, 2.54] |  |
| Poor lifestyle | 17/3905 | 1.83[0.76, 4.41] | 18/521388 | 2.23[1.05, 4.74] | 39/17186 | 2.11[1.32, 3.35] |  |
| **High genetic risk** |  |  |  |  |  |  |  |
| Optimal lifestyle | 10/32530 | 1.34[0.51, 3.53] | 16/802911 | 1.51[0.70, 3.26] | 72/3226055 | 2.15[1.42, 3.25] |  |
| Intermediate lifestyle | 15/38739 | 1.63[0.66, 4.01] | 24/633660 | 2.55[1.25, 5.21] | 61/2341088 | 2.38[1.56, 3.64] |  |
| Poor lifestyle | 8/13986 | 2.48[0.90, 6.84] | 5/18027 | 1.96[0.68, 5.66] | 16/6098 | 2.40[1.32, 4.36] |  |

Estimated HRs were based on the adjusted Cox proportional hazards regression models, with adjustment for age, socioeconomic status (index of multiple deprivation, fifth), family history of cancer and first 10 principal components of ancestry.

Abbreviation: CI, Confidence Interval.

| **Table S12.** Risk of incident bladder cancer according to genetic and lifestyle risk stratified by diabetes | | | | | |
| --- | --- | --- | --- | --- | --- |
|  | Non-diabetes | | Diabetes | | P for interaction |
|  | Cases/ Person-years | Hazard ratio (95% CI) | Cases/ Person-years | Hazard ratio (95% CI) |  |
| **Low genetic risk** |  |  |  |  | 0.1143 |
| Optimal lifestyle | 40/352388 | 1.00[Reference] | 1/10884 | 1.00[Reference] |  |
| Intermediate lifestyle | 74/396630 | 1.56[1.06, 2.30] | 3/25826 | 1.27[0.13, 12.26] |  |
| Poor lifestyle | 15/56565 | 2.30[1.27, 4.17] | 4/6175 | 7.62[0.85, 68.39] |  |
| **Intermediate genetic risk** |  |  |  |  |  |
| Optimal lifestyle | 152/1039128 | 1.30[0.92, 1.84] | 17/32404 | 5.65[0.75, 42.5] |  |
| Intermediate lifestyle | 247/1166379 | 1.79[1.28, 2.51] | 38/76793 | 5.54[0.76, 40.35] |  |
| Poor lifestyle | 50/164407 | 2.64[1.74, 4.01] | 12/18828 | 7.54[0.98, 58.14] |  |
| **High genetic risk** |  |  |  |  |  |
| Optimal lifestyle | 72/356852 | 1.79[1.22, 2.63] | 8/11141 | 7.98[1.00, 63.82] |  |
| Intermediate lifestyle | 100/404524 | 2.08[1.44, 3.00] | 20/27542 | 8.03[1.08, 59.91] |  |
| Poor lifestyle | 22/58216 | 3.40[2.02, 5.73] | 5/6358 | 8.65[1.01, 74.28] |  |

Estimated HRs were based on the adjusted Cox proportional hazards regression models, with adjustment for age, socioeconomic status (index of multiple deprivation, fifth), family history of cancer and first 10 principal components of ancestry.

Abbreviation: CI, Confidence Interval.

| **Table S13**. Hazard ratio (95% CI) of bladder cancer according to lifestyle category risk in sensitivity analyses | | | |
| --- | --- | --- | --- |
|  | Cases/ Person-years | Hazard ratio (95% CI) | P value |
| **Excluding incident cases during the first year of follow-up** |  |  |  |
| Poor lifestyle | 96/309836 |  |  |
| Intermediate lifestyle | 406/2094318 | 0.61[0.49, 0.76] | <0.0001 |
| Optimal lifestyle | 242/1800326 | 0.45[0.35, 0.57] | <0.0001 |
| Per SD |  | 0.76[0.70, 0.81] | <0.0001 |
| **Competing Risk Analysis using Fine-Gray subdistribution hazard model** |  |  |  |
| Poor lifestyle | 108/310549 |  |  |
| Intermediate lifestyle | 482/2097693 | 0.64[0.51, 0.80] | <0.0001 |
| Optimal lifestyle | 290/1802797 | 0.48[0.38, 0.61] | <0.0001 |
| Per SD |  | 0.77[0.72, 0.83] | <0.0001 |
| **Using a weighted lifestyle score** |  |  |  |
| Poor lifestyle | 298/885391.6 |  |  |
| Intermediate lifestyle | 509/2744670.3 | 0.55[0.47, 0.63] | <0.0001 |
| Optimal lifestyle | 73/580977.2 | 0.43[0.33, 0.55] | <0.0001 |
| Per SD |  | 0.73[0.69, 0.78] | <0.0001 |

Estimated HRs were based on the adjusted Cox proportional hazards regression models, with adjustment for age, sex, socioeconomic status (index of multiple deprivation, fifth), family history of cancer and first 10 principal components of ancestry, and genetic risk.

Abbreviation: CI, Confidence Interval; SD, standard deviation.

| **Table S14**. Associations lifestyle score and risk of bladder cancer stratified by PRS category in sensitivity analyses | | | | | | | |  |  |  |  |
| --- | --- | --- | --- | --- | --- | --- | --- | --- | --- | --- | --- |
|  | **Low genetic risk** | | |  | **Intermediate genetic risk** | | |  | **High genetic risk** | | |
|  | Cases/ Person-years | Hazard ratio (95% CI) | P trend value |  | Cases/ Person-years | Hazard ratio (95%CI) | P trend value |  | Cases/ Person-years | Hazard ratio (95% CI) | P trend value |
| **Excluding incident cases during the first year of follow-up** | | | 0.013 |  |  |  | <0.001 |  |  |  | 0.004 |
| Poor lifestyle | 16/62585 | 1.00[Reference] |  |  | 56/182814 | 1.00[Reference] |  |  | 24/64437 | 1.00[Reference] |  |
| Intermediate lifestyle | 62/421744 | 0.58[0.33, 1.00] |  |  | 238/1241188 | 0.62[0.46, 0.83] |  |  | 106/431386 | 0.60[0.39, 0.94] |  |
| Optimal lifestyle | 38/362747 | 0.44[0.24, 0.80] |  |  | 136/1070068 | 0.44[0.32, 0.60] |  |  | 68/367511 | 0.47[0.30, 0.76] |  |
| **Limiting the participants in white British descent** | | | 0.008 |  |  |  | <0.001 |  |  |  | 0.005 |
| Poor lifestyle | 16/56677 | 1.00[Reference] |  |  | 54/163658 | 1.00[Reference] |  |  | 26/58281 | 1.00[Reference] |  |
| Intermediate lifestyle | 69/382403 | 0.64[0.37, 1.11] |  |  | 261/1123886 | 0.70[0.52, 0.94] |  |  | 105/391507 | 0.56[0.36, 0.86] |  |
| Optimal lifestyle | 39/327615 | 0.45[0.25, 0.82] |  |  | 160/967210 | 0.53[0.39, 0.72] |  |  | 71/332400 | 0.46[0.29, 0.73] |  |
| **Competing Risk Analysis using Fine-Gray subdistribution hazard model** | | | 0.034 |  |  |  | <0.001 |  |  |  | 0.008 |
| Poor lifestyle | 19/ 62741 | 1.00[Reference] |  |  | 62/183235 | 1.00[Reference] |  |  | 27/64573 | 1.00[Reference] |  |
| Intermediate lifestyle | 77/422456 | 0.60[0.34, 1.06] |  |  | 285/1243172 | 0.65[0.48, 0.86] |  |  | 120/ 432065 | 0.63[0.40, 0.98] |  |
| Optimal lifestyle | 41/363272 | 0.47[0.26, 0.88] |  |  | 169/1071532 | 0.47[0.34, 0.64] |  |  | 80/367993 | 0.50[0.31, 0.80] |  |
| **Using a weighted lifestyle score** | |  | <0.001 |  |  |  | <0.001 |  |  |  | 0.007 |
| Poor lifestyle | 51/177500 | 1.00[Reference] |  |  | 180/526122 | 1.00[Reference] |  |  | 67/181770 | 1.00[Reference] |  |
| Intermediate lifestyle | 77/555264 | 0.49[0.34, 0.70] |  |  | 296/1625022 | 0.53[0.44, 0.64] |  |  | 136/564384 | 0.63[0.47, 0.85] |  |
| Optimal lifestyle | 9/115705 | 0.32[0.16, 0.66] |  |  | 40/346794 | 0.38[0.27, 0.54] |  |  | 24/118478 | 0.61[0.38, 0.98] |  |

Abbreviation: CI, Confidence Interval; SD, standard deviation.

Estimated HRs were based on the adjusted Cox proportional hazards regression models, with adjustment for age, sex, socioeconomic status (index of multiple deprivation, fifth), family history of cancer and first 10 principal components of ancestry.

| **Table S15.** Risk of incident bladder cancer according to genetic and lifestyle risk in sensitivity analyses | | | | | | |  |  |
| --- | --- | --- | --- | --- | --- | --- | --- | --- |
|  | Excluding incident cases during the first year of follow-up | | Limiting the participants in white British descent | | Competing Risk Analysis using Fine-Gray subdistribution hazard model | | Using a weighted lifestyle score | |
|  | Cases/ Person-years | Hazard ratio  (95% CI) | Cases/ Person-years | Hazard ratio  (95% CI) | Cases/ Person-years | Hazard ratio  (95% CI) | Cases/ Person-years | Hazard ratio (95% CI) |
| **Low genetic risk** |  |  |  |  |  |  |  |  |
| Optimal lifestyle | 38/362747 | 1.00[Reference] | 39/327615 | 1.00[Reference] | 41/363272 | 1.00[Reference] | 9/115705 | 1 [Reference] |
| Intermediate lifestyle | 62/421744 | 1.32[0.88, 1.98] | 69/382403 | 1.43[0.96, 2.11] | 77/422456 | 1.43[0.96, 2.11] | 77/555264 | 1.56[0.78, 3.10] |
| Poor lifestyle | 16/62585 | 2.37[1.32, 4.26] | 16/56677 | 2.28[1.27, 4.08] | 19/62740 | 2.28[1.27, 4.08] | 51/177500 | 3.23[1.59, 6.56] |
| **Intermediate genetic risk** |  |  |  |  |  |  |  |  |
| Optimal lifestyle | 136/1070068 | 1.23[0.86, 1.76] | 160/967210 | 1.40[0.99, 1.99] | 169/1071532 | 1.40[0.99, 1.99] | 40/346794 | 1.50[0.73, 3.10] |
| Intermediate lifestyle | 238/1241188 | 1.74[1.24, 2.45] | 261/1123886 | 1.86[1.33, 2.61] | 285/1243171 | 1.86[1.33, 2.61] | 296/1625022 | 2.05[1.06, 3.99] |
| Poor lifestyle | 56/182814 | 2.82[1.86, 4.26] | 54/163657 | 2.67[1.76, 4.03] | 62/183235 | 2.67[1.76, 4.03] | 180/526122 | 3.89[1.99, 7.61] |
| **High genetic risk** |  |  |  |  |  |  |  |  |
| Optimal lifestyle | 68/367511 | 1.78[1.2, 2.65] | 71/332400 | 1.80[1.22, 2.66] | 80/367993 | 1.80[1.22, 2.66] | 24/118478 | 2.63[1.22, 5.66] |
| Intermediate lifestyle | 106/431386 | 2.21[1.53, 3.21] | 105/391507 | 2.13[1.47, 3.08] | 120/432065 | 2.13[1.47, 3.08] | 136/564384 | 2.70[1.38, 5.31] |
| Poor lifestyle | 24/64437 | 3.54[2.12, 5.92] | 26/58281 | 3.73[2.27, 6.13] | 27/64573 | 3.73[2.27, 6.13] | 67/181770 | 4.21[2.10, 8.44] |

Abbreviation: CI, Confidence Interval; SD, standard deviation.

Estimated HRs were based on the adjusted Cox proportional hazards regression models, with adjustment for age, sex, socioeconomic status (index of multiple deprivation, fifth), family history of cancer and first 10 principal components of ancestry.

| **Table S16.** The joint effect of genetic and lifestyle factors on early-and late-onset of bladder cancer risk | | | | | |
| --- | --- | --- | --- | --- | --- |
|  | Cancer diagnosed in adults <65 years | |  | Cancer diagnosed in adults >65 years | |
|  | Cases/ Person-years | Hazard ratio (95% CI) |  | Cases/ Person-years | Hazard ratio (95% CI) |
| **Low genetic risk** |  |  |  |  |  |
| Optimal lifestyle | 8/120089.59 | 1.00[Reference] |  | 33/243182.22 | 1.00[Reference] |
| Intermediate lifestyle | 14/145440.67 | 1.31[0.55, 3.13] |  | 63/277015.47 | 1.56[1.02, 2.38] |
| Poor lifestyle | 5/23765.75 | 2.66[0.87, 8.17] |  | 14/38974.92 | 2.51[1.34, 4.69] |
| **Intermediate genetic risk** |  |  |  |  |  |
| Optimal lifestyle | 32/357586.53 | 1.38[0.63, 2.99] |  | 137/713945.53 | 1.42[0.97, 2.08] |
| Intermediate lifestyle | 75/426948.36 | 2.40[1.16, 4.99] |  | 210/816223.36 | 1.79[1.24, 2.59] |
| Poor lifestyle | 19/69698.67 | 3.17[1.38, 7.28] |  | 43/113536.33 | 2.61[1.65, 4.11] |
| **High genetic risk** |  |  |  |  |  |
| Optimal lifestyle | 20/122602.33 | 2.63[1.16, 5.97] |  | 60/245390.78 | 1.81[1.18, 2.76] |
| Intermediate lifestyle | 27/147646.75 | 2.45[1.11, 5.41] |  | 93/284418.49 | 2.27[1.52, 3.37] |
| Poor lifestyle | 8/24896.64 | 3.87[1.44, 10.36] |  | 19/39676.77 | 3.42[1.94, 6.02] |

Estimated HRs were based on the adjusted Cox proportional hazards regression models, with adjustment for age, sex, socioeconomic status (index of multiple deprivation, fifth), family history of cancer and first 10 principal components of ancestry.

Abbreviation: CI, Confidence Interval; SD, standard deviation.


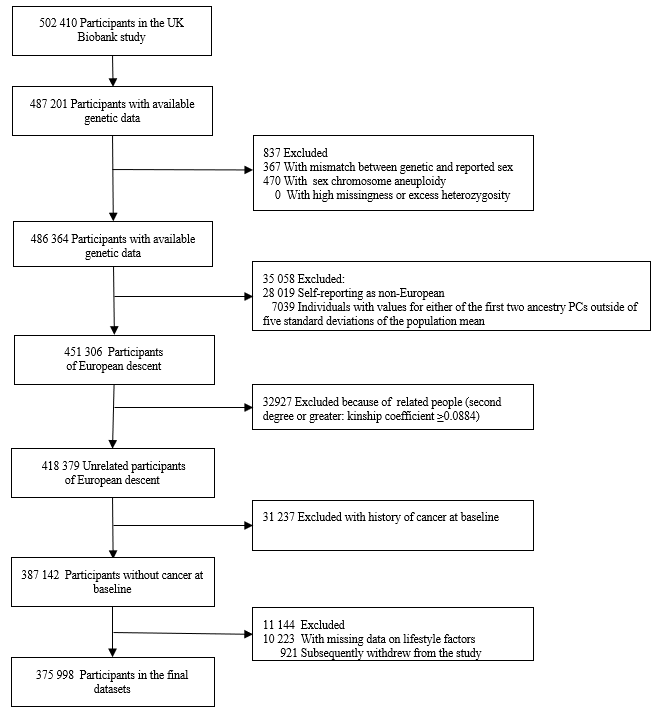


**Figure S1.** Flowchart for the selection of the study population from UK Biobank study


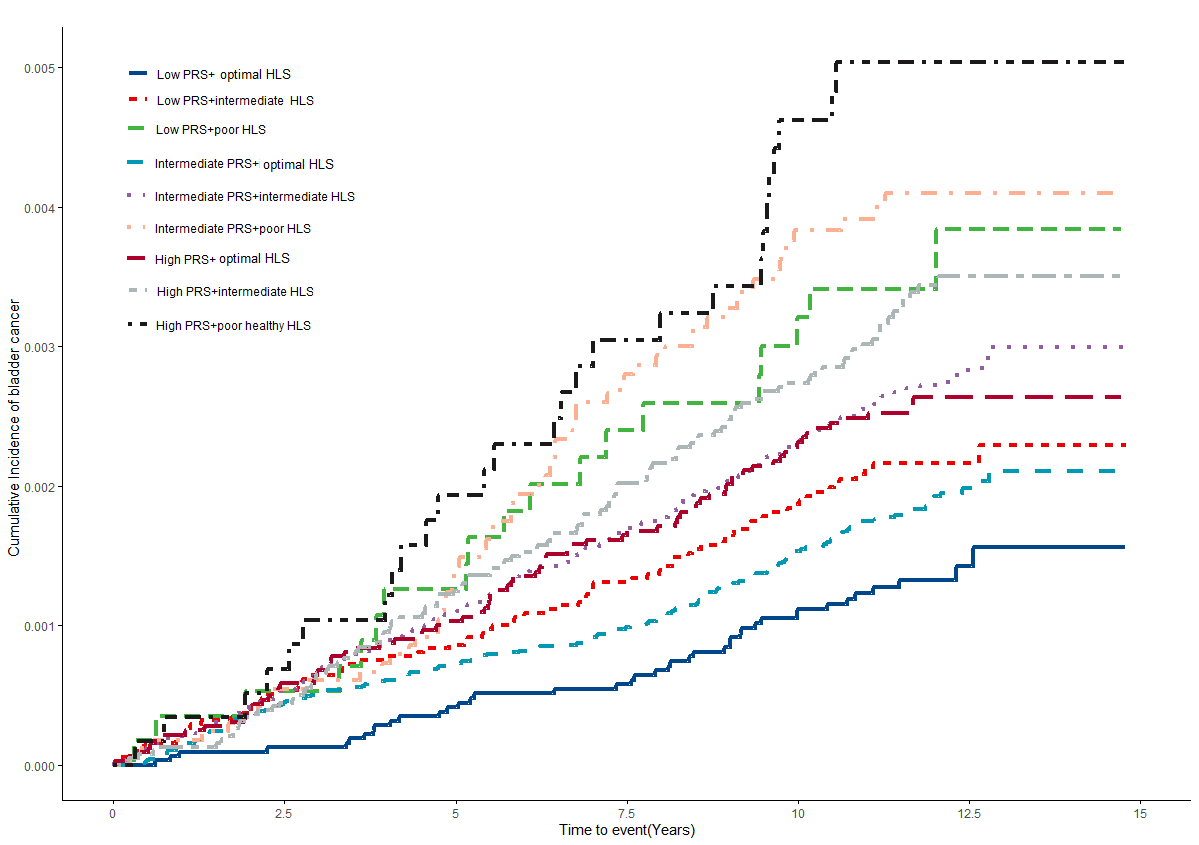


**Figure S2.** Risk of incident bladder cancer according to genetic risk and lifestyle factors

Note: HLS, healthy lifestyle score; PRS, polygenic risk score.


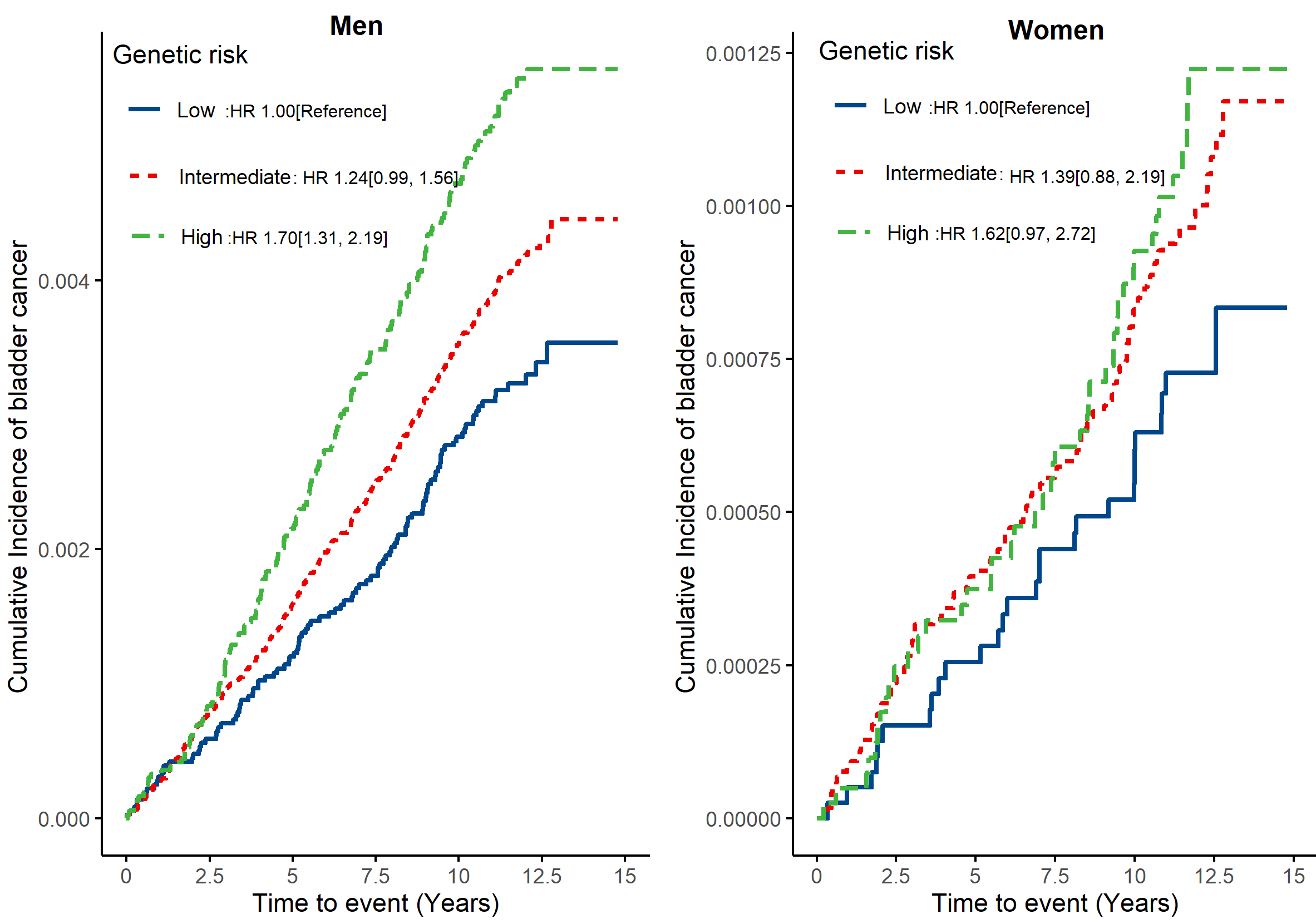


**Figure S3.** Risk of incident bladder cancer according to genetic risk stratified by sex


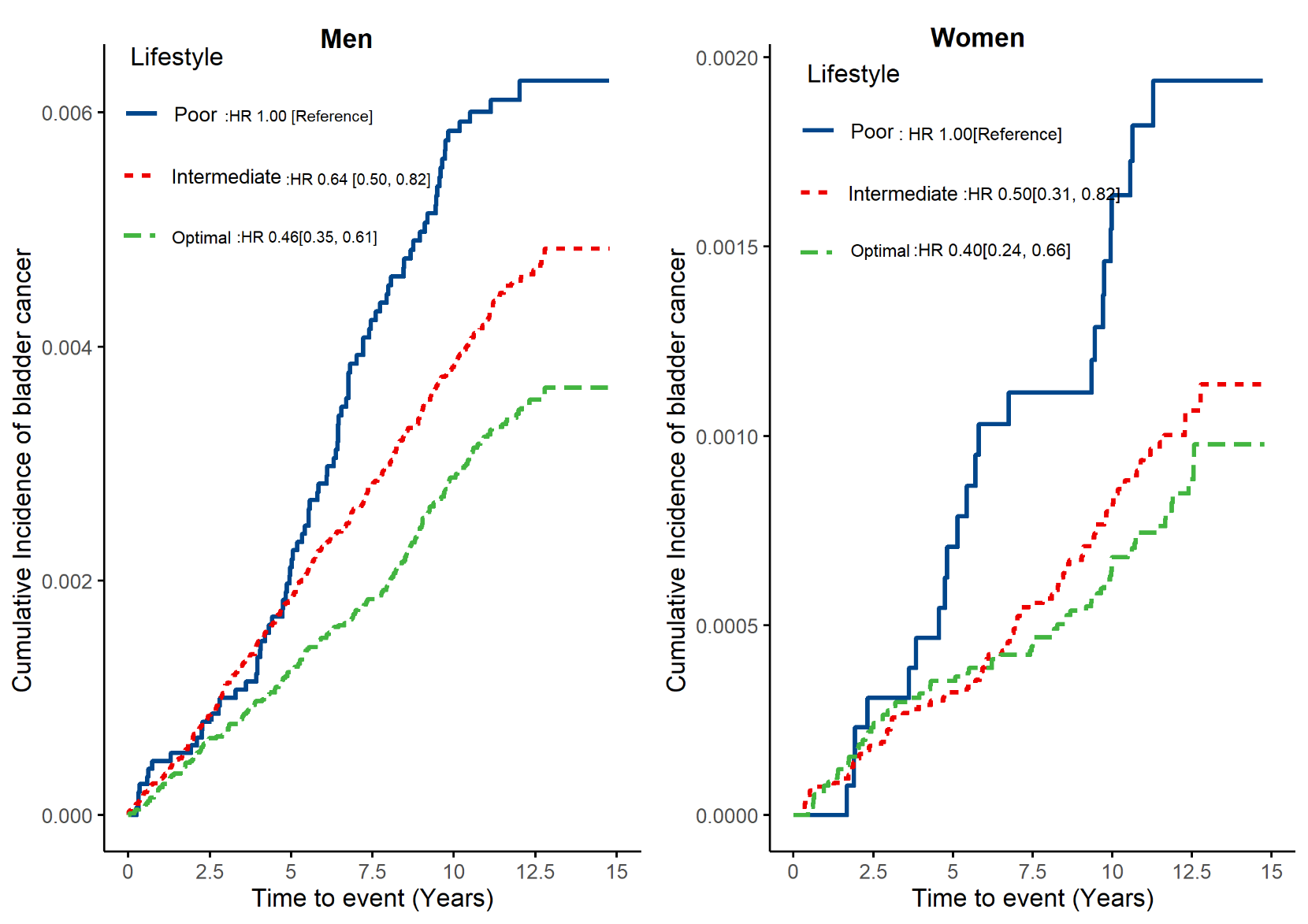


**Figure S4.** Risk of incident bladder cancer according to lifestyle profile stratified by sex


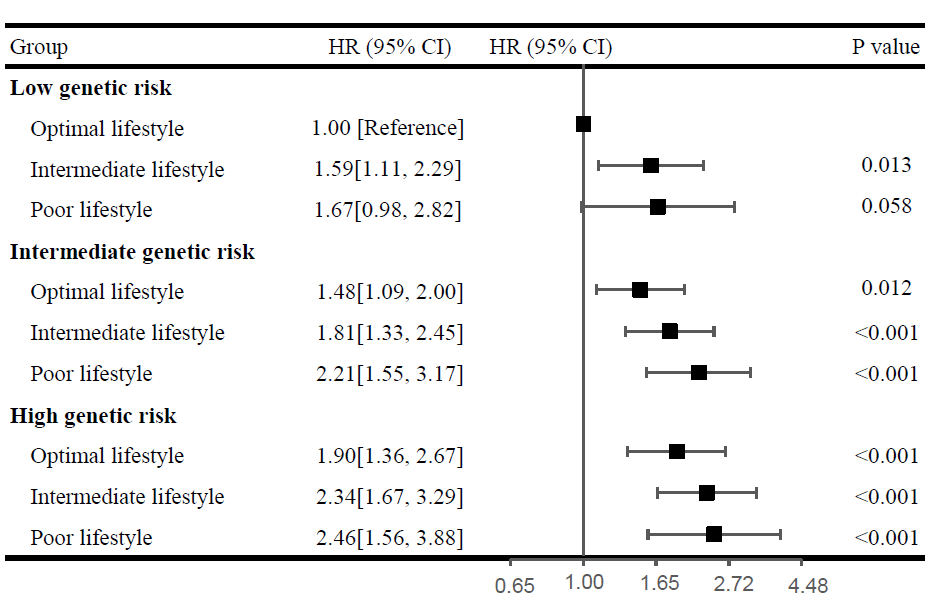


**Figure S5.** Risk of incident bladder cancer according to genetic and lifestyle risk (without smoking status)
